# Cost-Effectiveness of Influenza Vaccination in Healthy Children: A 10-Year Population-Based Study

**DOI:** 10.3390/vaccines12101113

**Published:** 2024-09-28

**Authors:** Elisa Barbieri, Yuxi Wang, Anna Cantarutti, Antonio Scamarcia, Luigi Cantarutti, Giovanni Corrao, Aleksandra Torbica, Carlo Giaquinto

**Affiliations:** 1 Division of Pediatric Infectious Diseases, Department of Women’s and Children’s Health, University of Padua, 35100 Padua, Italy; 2 Centre for Research on Health and Social Care Management, Bocconi University, 20136 Milan, Italy; 3 Institut National D’études Démographiques, 75980 Paris, France; 4 Unit of Biostatistics, Epidemiology and Public Health, Department of Statistics and Quantitative Methods, University of Milano-Bicocca, 20126 Milan, Italy; 5 National Centre for Healthcare Research and Pharmacoepidemiology, Department of Statistics and Quantitative Methods, University of Milano-Bicocca, 20126 Milan, Italy; 6 Società Servizi Telematici—Pedianet, 35100 Padua, Italy; 7 Department of Social and Political Science, Bocconi University, 20136 Milan, Italy

**Keywords:** influenza vaccination, cost-effectiveness, children, Italy

## Abstract

**Background/Objectives**: Seasonal influenza annually puts a significant burden on the pediatric population, especially the youngest, causing severe illness and death. Additionally, associated healthcare costs cause a significant financial strain on healthcare systems. While vaccination is the most effective prevention method, its cost-effectiveness for healthy children remains unassessed. **Methods**: Using the Pedianet database spanning from 2009 to 2019, we analyzed influenza cases among 6-month-olds to 14-year-olds in Italy. Data included influenza-related medical visits, prescriptions, exams, emergency visits, hospitalizations, and costs. Adverse events and quality-adjusted life years (QALYs) were considered from the existing literature. A static decision-tree model compared annual vaccination strategies, assessing probabilities for influenza or influenza-like illnesses by vaccination status. Incremental cost-effectiveness ratios (ICERs) were calculated, along with sensitivity analyses and cost-effectiveness acceptability curve generation. **Results**: Mean total influenza costs for vaccinated children averaged EUR 18.6 (range 0–3175.9, including EUR 15.79 for the influenza vaccination), whereas costs for unvaccinated children were consistently lower at around EUR 4.6 (range 0–3250.1). The average ICER for years where vaccine and virus strains are matched was EUR 29,831 per QALY, which is below the EUR 40,000 threshold set by the Italian National Health Services. The ICER values range from EUR 13,736 (2017/2018) to EUR 72,153 (2013/2014). Averted influenza costs averaged EUR 23 per case, with fluctuations over the years. In most observed years, influenza vaccination was cost-effective from the healthcare providers’ standpoint. The exception was 2009–2010, due to a mismatch between vaccine and virus strains. **Conclusions**: This study highlights the economic viability of influenza vaccination, especially when virus and vaccine strains align. It demonstrates the potential of vaccination programs in preserving children’s health and well-being while managing healthcare costs.

## 1. Introduction

Annually, the seasonal influenza virus takes a profound toll on the pediatric demographic, with substantial morbidity, significant mortality, and an extensive financial burden reverberating through healthcare systems and society [1,2]. This necessitates not only ongoing research and development for effective prevention and treatment strategies but also the implementation of evidence-based public health measures to mitigate the multifaceted impact of this insidious virus.

Children are more vulnerable to serious complications from influenza. Indeed, before the COVID-19 pandemic, 6.2% of children under five years of age living in a high-income country had influenza each season, and around 111 out of 1000 hospital admissions in children were associated with influenza [3]. Moreover, influenza sequelae and complications, including acute otitis media, pneumonia, seizures, encephalopathy/encephalitis, myelitis, meningitis, focal neurologic deficit, Guillain–Barré syndrome, and death, were observed in previously healthy children and in those with an underlying condition, further increasing the clinical, societal, and economic burden of influenza disease [4,5,6,7,8,9,10].

Typically, direct costs associated with the treatment of influenza include primary-care consultations, medications, diagnostic tests and examinations, emergency room (ER) visits, and hospitalizations. Even if general practitioner (GP) consultations and prescriptions for medication are associated with small unit costs in most of the recent studies conducted in Europe (i.e., GP costs: ranging from EUR 32 in 2008 Italy to EUR 112 in 2014 in Finland; medications costs ranging from EUR 4 for antibiotics in 2008 in Italy to EUR 27 for antivirals and EUR 10 for antibiotics in 2012 in Germany), they account for a large proportion of the overall direct costs [10,11,12,13,14,15,16,17]. 

On the other hand, hospitalization costs represent the highest direct unit costs for influenza in children in most European countries, (ranging from a minimum of GBP 1050 in 2017 in England to a maximum of EUR 4467 in 2014 in France, according to a recent systematic review), accounting only for a minimum of the overall direct costs [17,18]. 

In the pediatric population, the burden of influenza disease is often imposed on the whole family, with parents facing indirect costs, including co-payments for physician office visits, prescription medications, and inpatient services, in areas with no healthcare systems free-of-charge at the point of delivery, as well as costs of self-medicating with over-the-counter drugs and production losses due to missed workdays when parents need to take time off to care for their sick child. A study conducted in Italy in the winter season of 2008–2009 estimated that the indirect costs of working days lost by parents (EUR 70) have the greatest impact on the average total cost of an influenza case in children [10].

Furthermore, the economic impact extends to society as a whole, with decreased school attendance and potential disruptions in childcare, leading to lost educational opportunities and productivity. 

The most effective way to prevent influenza and influenza-related complications is vaccination. A recent study assessed the effectiveness of the influenza vaccine (IV) in a cohort of healthy children in Italy from 2010 to 2019. The findings showed high IV effectiveness, ranging from 16% in the 2009–2010 season to 72% in the 2016–2017 season. However, in the season before the COVID-19 pandemic, only 9% of children were vaccinated against influenza [18].

Since 2020, different regions in Italy have started offering the IV free of charge to healthy children from six months up to six years of age. The main driver was the possibility of reducing the co-circulation of SARS-CoV-2 and influenza virus among the pediatric and adult population, given the risk of a high transmission rate in children because of prolonged viral shedding (i.e., individuals who may not yet be experiencing any of the viral symptoms are shedding viral particles while they talk, exhale, eat, and perform other normal daily activities) [19]. To the best of our knowledge, no comprehensive analysis was conducted to assess the cost-effectiveness of offering the IV free of charge to children in Italy. 

A recent systematic review evaluating the cost-effectiveness of influenza vaccination for the pediatric population in Europe found that extending pediatric vaccinations to the whole population using a live vaccine, especially in the quadrivalent formulation, is cost-effective compared to current vaccination policies (at-risk groups) with the trivalent influenza vaccine or no vaccination. The incremental cost-effectiveness ratio (ICER) ranged from GBP 298 per QALY to GBP 7989 per QALY, in all cases being below the threshold considered (GBP ~20,000) [20]. However, most studies used mathematical modelling or small-scale studies to derive the epidemiological burden for influenza with data imputed from the literature or expert consensus; few studies relied on real-world data from pediatricians, hospitals, or other administrative sources [21,22,23]. Therefore, if the vaccine efficacy and duration of immunity is estimated to be constant between seasons, the influenza epidemic would seem the same in size, but in reality, these parameters may vary over time. Increased variability in epidemic size may reduce the impact of the pediatric vaccination program, reducing the overall QALY loss as previously postulated in other studies [15,24].

Our research makes a valuable contribution to the scientific literature by presenting novel empirical evidence on the cost-effectiveness of expanding influenza vaccination programs to include all healthy children, using a population-based approach. This approach allows us to evaluate the collective impact and benefits of widespread vaccination within the entire pediatric population from the perspective of the healthcare provider. 

Regarding policy relevance, our study is one of the few that adopts the perspective of the entire healthcare system (i.e., both outpatient and inpatient settings). Our research findings shed light on the potential economic advantages of implementing widespread vaccination strategies for healthy children, offering evidence-based insights to inform public health policies and decision-making processes. Furthermore, by quantifying the cost-effectiveness of scaling up vaccination efforts, we contribute to the understanding of resource allocation within healthcare systems and provide a foundation for further investigations into optimizing influenza prevention strategies for the benefit of both children and the broader community.

## 2. Methods

### 2.1. Study Context: Italian National Health Service

The Italian National Health Service (NHS) is a publicly funded healthcare system that provides comprehensive medical services to all Italian residents. Established in 1978, the Italian NHS aims to ensure universal access to healthcare, guaranteeing quality services and equal treatment for all citizens. It operates under the principles of solidarity, equity, and universality. The Italian NHS covers a wide range of healthcare services, including primary care, hospital care, specialist consultations, emergency services, preventive care, and pharmaceutical assistance. The system is funded through general taxation and employee and employer contributions. 

The NHS plays a crucial role in implementing vaccination policies to safeguard public health. Italy has a comprehensive immunization program to protect its population from vaccine-preventable diseases. It strongly recommends annual influenza vaccination for all eligible children. The vaccination campaign specifically targets children aged 6 months to 16 years, aiming to protect their health and prevent the spread of influenza within schools and communities. Influenza vaccination services were readily available to children through family pediatricians participating in the vaccination campaign and designated vaccination centers. The Italian NHS emphasizes the importance of timely vaccination, typically recommending that children receive the influenza vaccine before the start of the flu season. Still, before 2020, the influenza vaccination was offered free of charge at the point of delivery only to a part of the population with differences between regions (i.e., people older than 65 years of age, people with comorbidities, and all those at higher risk) [25,26].

### 2.2. Database

Our study relies on the clinical records from the Pedianet database (http://www.pedianet.it, accessed on 1 March 2022). The Pedianet database in Italy is a nationwide pediatric primary-care database that collects and analyzes comprehensive data on children’s health. The database consists of anonymized electronic health records from participating family pediatricians (FPs), encompassing a large and diverse population of children. The Pedianet database captures a wide range of information, including demographic data, medical diagnoses, prescriptions, vaccinations, and laboratory results.

Individual children were included if (i) they were followed by one of the FPs of the Pedianet network adhering to the flu vaccination program (i.e., FPs who actively vaccinated against influenza during the influenza season in agreement with the National Healthcare System), (ii) have been enrolled in Pedianet for at least one year and had at least two outpatient encounters (except those aged less than 12 months), and, (iii) were aged between 6 months to 14 years during the observation period. Children with comorbidities were excluded. Exposed children were all children who received the influenza vaccine during at least one influenza season. The reference group consisted of children who had never received the IV during the same period [18].

### 2.3. Vaccine Effectiveness and Health Outcomes

Vaccine effectiveness against influenza is retrieved from previous work assessing the effectiveness of influenza vaccine using a Cox proportional-hazards model adjusted for age, gender, and region of birth [18]. The hazard ratios and 95% confidence intervals were estimated for each influenza season of interest, showing that the IV was effective in preventing influenza or ILI in healthy children. The effectiveness rates for each year are shown in Table S1 in the Supplementary Materials. Due to the considerable variation in effectiveness that can be attributed to the influenza strain circulation mismatch, we have summarized the influenza circulation lineage and matching in Italy over the observation period (Table S2). The interpretations of the vaccine’ effectiveness should go together with the matching information.

An important health outcome for the economic analysis is the quality-adjusted life years (QALYs) differences due to occurrences of influenza. The unit QALY measures from different clinical pathways are obtained from the existing literature [26,27]. Specifically, assuming a baseline utility of 0.95, disutility derived from a single influenza episode without a specialist or hospital visit is around 0.009, disutility derived from a specialist visit is about 0.0045, and disutility derived from hospitalization is about 0.0112. The utility value is obtained using population-based values of the EuroQol five-dimensional (EQ-5D) questionnaire in Italy, while the QALY lost due to the different clinical pathways undertaken are obtained from a study on health-related quality of life using the EQ-5D questionnaire in Spain [26,27]. All the parameters and sources can be found in Table S4.

### 2.4. Episode Definition, Healthcare Resources Utilization, and Costs Estimation

To define the healthcare resources, we considered all family pediatrician visits, ambulatory diagnostics exams, laboratory or specialists’ exams, and ER visits that (i) are related to the influenza episodes, (ii) precede the diagnosis index date by 7 days and fall within 30 days from the index date of an outpatient influenza episode, and (iii) influenza or influenza complication is one of the diagnoses. In cases of hospitalization, the episode end date is calculated 30 days from the hospital discharge date.

All “well-child visits” (which are performed routinely by FPs based on the child’s age) with no mention of influenza were included in the healthcare resource utilization assessment. Ambulatory diagnostics exams included the influenza rapid test, the C reactive protein rapid test, the urine stick, and oxygen measurement. Moreover, for complications, we considered the pneumatic otoscopy exam date (for acute otitis media (AOM) and the impedenzometer exam date (for AOM). Laboratory or specialist exams included are polymerase chain reaction (PCR) tests prescription date, complete blood exams prescription, microbiological prescription, and pneumologist visits prescription. More details on the complications due to influenza can be found in the Supplementary Materials.

A medical prescription was considered related to the influenza episodes if it fell within 30 days from the index date of the influenza episode. In Italy, medicines are sold prepackaged in specific quantities. The number of packages for outpatient prescriptions are known and reported in the database. Antibiotics prescribed during an FP visit where a concomitant bacterial infection was reported were not considered. The same applies to ongoing antibiotic therapy (i.e., an antibiotic therapy prescribed in the 14 days preceding the influenza start date). Prescriptions were retrieved and grouped based on the ATC code; medications considered include antibiotics (J01*), antivirals (J05*), and medicine acting on the respiratory tract (R03*). More details on the medicine can be found in Table S3.

To identify the unit costs that are not observed in our sample, we considered both fixed and variable costs related to pediatric influenza and complications. The costs of pediatric visits, medicines, and lab tests were calculated using the prescribing information and administrative information from Pedianet and information from ER and hospitalization discharge letters. Fixed costs include vaccination and administration of the IV. Since the management of the IV is not uniform among the various Italian regions (in some regions, the IV is administered by the FPs in the ambulatory setting while in other regions by healthcare specialists in the vaccination centers) and because the cost of administering the IV is based on regional agreements, this cost was calculated as a simple average between the maximum value (approximately EUR 15 if carried out by the FPs) and the minimum value (approximately EUR 6 if carried out by the vaccination center) of the cost that is assumed to be associated with the administration of the influenza vaccine in Italy, for an estimate of EUR 10. A price of EUR 5.79 per unit per IV was used based on the literature [28]. For the visit to the FP, a cost of EUR 20.66 was assumed, deriving from the tariff nomenclator for the specialist visit. Hospitalization costs due to influenza were retrieved from the literature [29], while all other unitary cost measures were estimated from the dataset (see Table S4 in the Supplementary Materials).

### 2.5. Cost-Effectiveness Analysis

Cost-effectiveness analysis (CEA) reveals the trade-offs involved in choosing among alternative interventions to obtain the most health possible for the available resources. The analysis defines the potential loss of health benefits relative to the associated healthcare resource costs if the pediatric IV is not adopted [30]. Because influenza causes annual epidemics and yearly vaccination was implemented, we use a time horizon of one vaccination season each year for the 10-year observation period without discount rates. 

Following the established guideline [31,32,33], we developed a decision-tree model that defined two strategies: vaccinating or not vaccinating children each influenza season. We use a static model because (i) we lack important data inputs such as contact rates, age-specific transmission probabilities, and recovery rates for the pediatric population, and (ii) the estimates from static models are more conservative [34]. 

Both the “vaccination” and “no vaccination” branches are further divided depending on whether influenza or influenza-like illness develops, because vaccinated children may or may not be protected; as seen in Table S1, the effectiveness ranges from 16% to 73% depending on the season and IV strain matching with the circulating virus. For the symptomatic children, we further divided the population into whether the patient sought specialist and/or hospital care. We estimated the annual probabilities of developing symptomatic influenza and using different medical services by vaccination status using the Pedianet dataset. The average probability of paying a pediatric visit without further specialist or hospital visits was around 9% for unvaccinated and 5% for vaccinated patients. The probability of going to specialist visit was also higher for unvaccinated children. 

For each influenza season, we obtained the incremental cost-effectiveness ratios (ICERs) to delineate the results of the analysis. The ICER was calculated by dividing the difference in costs by the difference in QALYs. Because children are not part of the labor force, we carried out the analysis from the provider perspective, only considering the direct benefit and cost of administering the IV to children. We also do not use discount rates because we conduct a yearly analysis on the immediate effect of the vaccines. The findings of the cost-effectiveness analysis should provide the same information as used for making decisions about any purchasing choice. More detailed inputs are in Table 1. This part of the analysis is carried out using Excel.

### 2.6. Sensitivity Analysis

We developed both a one-way deterministic sensitivity analysis (DSA) as well as a probabilistic sensitivity analysis (PSA) from the healthcare provider’s perspective to test how the cost-effectiveness of testing varies according to parameter changes. For the DSA, we vary the probability parameters by 25% to assess how sensitive our ICER values are to the estimated values. Using a total of 1000 Monte Carlo simulations, we varied the QALY (beta distribution) and cost (gamma distribution) to generate the cost-effectiveness acceptability curve (CEAC), which presents the probability of being cost-effective over the different thresholds. The CEAC is compared with the maximum willingness-to-pay (WTP). The WTP per year of life lost is a theoretical maximum price that a healthcare system is willing to pay to prevent the loss of one year of quality-adjusted life. Health economists have conducted studies to infer that in Italy the willingness to pay threshold is between EUR 25,000 (minimum) and EUR 40,000 (maximum) [33]. The DSA is performed on Excel, while the PSA is computed using STATA 17.

## 3. Results

**Figure 1:**
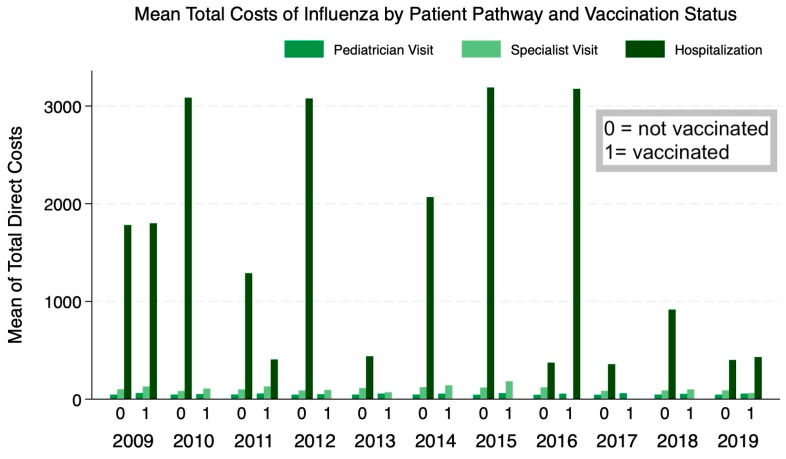
Average total costs of influenza/ILI by vaccination status and influenza season. Pedianet, 2009–2019.

**Figure 2:**
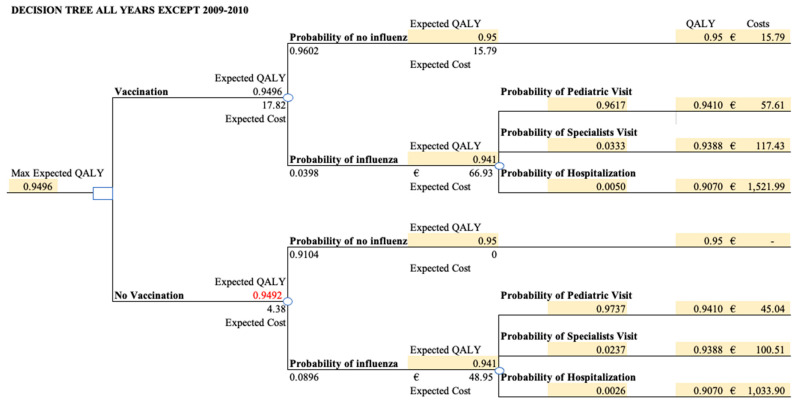
Sample of the static decision-tree model defining the two strategies each season: vaccinating or not vaccinating children.

We first show in Figure 1 the distribution of total observed costs incurred due to an influenza episode by the patient pathway, vaccination status, and across influenza seasons. We notice that for most influenza seasons, there are no hospitalization costs given the effectiveness of the vaccine, but the average cost of pediatrician and specialist visits remains similar across vaccinated and unvaccinated children. After incorporating the probabilities of patient pathways, we derived the expected total influenza costs per patient for vaccinated children at around EUR 17.82 (including vaccination estimated to be equal to EUR 15.79), while the expected total influenza costs for unvaccinated children are around EUR 4.39 (Table 2). The mean cost and the probabilities of each patient pathway are further inputted into the decision tree for each season for the calculation of incremental values. In Figure 2, we illustrate a sample decision tree for the values obtained for the 2017–2018 season.

As mentioned, the expected QALY and costs were calculated using the average unit values from Table 2 and the probabilities calculated from the dataset. We calculated the incremental QALY gains from vaccination for all seasons as the primary clinical outcome (see Table 3). The QALY gain per vaccinated child is on average 0.00045 life years, with the 2017–2018 reaching the highest benefit. We estimated the expected healthcare expenditure for each influenza season. The expected QALY is higher under vaccination because the probability of influenza onset is much lower. However, the unit costs are also higher for vaccinated children because, even with the lower probability of influenza, these children all incur a fixed vaccination cost. The incremental cost of vaccination is around EUR 15 on average across all seasons, with no substantial variations (Table 2).

**Figure 3:**
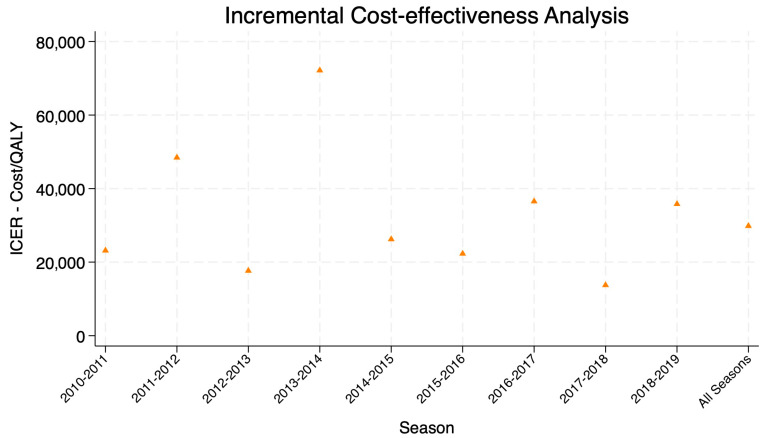
Incremental cost-effectiveness analysis by influenza season.

For the primary outcomes for our CEA, we calculated the ICERs per QALY gained for each season to understand the cost and effectiveness trade-off for the vaccination program. As seen in Figure 3, the average ICER for matched seasons is estimated to be around EUR 29.831 per QALY, and varies greatly across season, from EUR 13,736 in 2017/2018 to EUR 72,153 in 2013/2014. We also estimated the cost per influenza case averted using the previous study on vaccine effectiveness, which was around EUR 23 per case, with some fluctuations across the years. Figure 3 visualizes the ICERs across all seasons except 2009–2010. From the healthcare system’s perspective, the inclusion of pediatric vaccination is expected to incur higher direct costs but remain cost-effective to the threshold of EUR 40,000 per QALY for most of the observed period. The exact values of ICERs can be found in Supplementary Materials Table S4.

**Figure 4:**
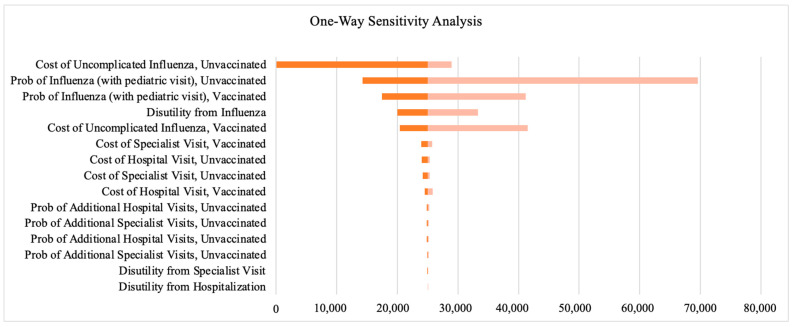
Tornado plot of deterministic one-way sensitivity analysis.

For the sensitivity analysis, we first assessed how different factors affect the ICER values, keeping everything else constant. The tornado graph (Figure 4) shows that ICER is most sensitive to the direct costs of treating unvaccinated influenza cases, followed by vaccine effectiveness and disutility derived from influenza.

**Figure 5:**
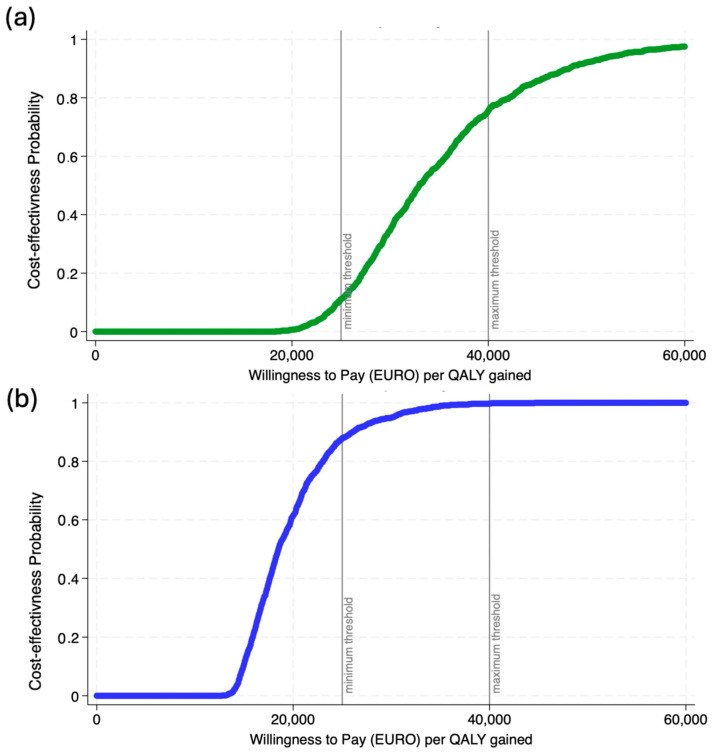
Probabilistic sensitivity analysis: cost-effectiveness acceptability curve, (**a**) all seasons [except 2009–2010] and (**b**) 2017–2018 season.

We also created the CEACs where the threshold values of willingness to pay were plotted against the portion of simulations that resulted in ICERs falling below the threshold. We did so for both the average values (except for 2009–2010), and the season influenza vaccines were most cost-effective (2017–2018). As shown in Figure 5a, vaccinating children against influenza had about a 75% probability of being cost-effective across all observed seasons, while it had an almost 100% probability of being cost-effective for the 2017–2018 influenza season (Figure 5b).

## 4. Discussion

Our study utilized real-world data, combining primary-care information with administrative records, to evaluate the cost-effectiveness of vaccinating children against influenza. Our findings demonstrated that influenza vaccination programs (IV programs) are highly cost-effective from a healthcare provider standpoint, with an average incremental cost-effectiveness ratio (ICER) of approximately EUR 29,831 per quality-adjusted life year (QALY). Notably, the ICER per QALY remained below the widely accepted threshold of EUR 40,000, as commonly employed by the Italian National Health Services, for all years except 2009–2010 when there was a vaccine–strain mismatch.

The static model that we used is more conservative compared to the dynamic model that can be used to model cost-effectiveness of influenza vaccines. Indeed, the dynamic model takes into account the proportion of the vaccinated population, the burden of the disease, and the possible “herd immunity” [35]. The “herd immunity” effect could lead to a decrease in influenza cases among high-risk groups in the same age class as well as other susceptible ones such as the elderly (those in contact with children such as grandchildren), thereby mitigating the overall burden of the disease and improving cost-effectiveness from a societal perspective. 

A major strength of our study was the use of the Pedianet primary-care database with information on ER and hospitalization, which allowed us to evaluate the impact of the IV on influenza/ILI episodes in the overall pediatric population cared for in the outpatient and inpatient settings. Because of this, we derived a cost-effectiveness analysis using real-world data instead of simulations. Indeed, our findings report a lower ICER value per QALY than results from the study by Mennini et al., (EUR 29,831 on average vs. EUR 110,083 in children 4 months to 4 years of age and EUR 148,021 for those aged 5–19 years) [21], where VE in children was modelled from adult data considering the whole pediatric population, not just healthy subjects. Second, given the extent of the historical data, we estimated the CEA over 10 influenza seasons. We were indeed able to derive the CEA for each season with a high match of the serotypes included in the IV with the circulating viruses and for each season with a low match (i.e., the 2009–2010 season). This is of particular importance because data used in CEA analysis are usually limited to one season, while in the real world, epidemics due to particular virulent strains have happened, limiting the VE and varying the infection rate as well as the clinical burden [36]. Indeed, giving that occasional mismatch can happen, pooling data from different seasons might be more informative to health policy makers implementing an annual universal vaccination policy.

Our study also had several limitations. The first limitation concerns the retrospective nature of the study, which allowed us to rely only on the information reported by FPs. Hence, we cannot exclude that some influenza episodes or complications were not reported as well as some unmeasured clinical features of the children. However, because medical care is needed in case of an ILI/influenza episode, children would likely be visited by the FP. Moreover, before 2020, FPs were in charge of granting the illness certificate to allow caregivers to be exempted from work to take care of the child [37]. Second, we excluded children enrolled by FPs who did not adhere to the regional vaccination campaign. This, even if it allowed us to increase the data quality by reducing the misclassification of the exposure because FPs receive a reimbursement for every vaccine administered, could have affected the representativeness of the study cohort, especially in terms of regional representativeness. Third, we based our outcome on the influenza/ILI diagnosis derived from the clinical assessment of the FPs rather than a laboratory confirmation, which might be subjective. Fourth, residual confounding might be present. In the vaccine effectiveness analysis used as a reference, despite the fact that the authors used propensity score stratification to balance the vaccinated and non-vaccinated populations, no further method was adopted to detect residual confounding (i.e., such as negative control—an outcome unexpected to be affected by the exposure of interest- or off-season outcomes—estimating VE outside of the influenza season, when a vaccine effect should be absent). This residual confounding might have resulted in an increased or decreased estimation of effectiveness. To account for this, we have performed sensitivity analyses. Fifth, because of insufficient national data, we assumed that the estimated QALYs lost due to influenza would be the same as in a previous study conducted in the UK [11]. Despite this limitation, the one-way sensitivity analysis in our model showed that this parameter did not result in significant variation in the ICER. Sixth, we did not stratify the analysis based on the effectiveness of the type of vaccine (i.e., trivalent versus quadrivalent or inactivated versus live attenuated) because of missing information. Still, inactivated influenza vaccines are indicated as a primary target, with live attenuated ones indicated in older children to achieve the best vaccination coverage [38,39,40]. Finally, we chose to study the healthcare payer prospective considering direct costs only because children are not part of the labor force. However, it is true that caregivers also incur costs related to days of work lost and out-of-pocket medications. Indeed, a prospective study conducted in collaboration with FPs of the Pedianet network during the influenza season of 2007–2008, assessed indirect costs related to caregivers’ days of work lost to be between EUR 48 and EUR 90, with values even higher for persons taking care of preschoolers [10].

## 5. Conclusions

The cost-effectiveness of influenza vaccination programs from a healthcare provider perspective hinges on the alignment between the circulating virus and vaccine strains. Additional data must consider variables such as the specific influenza vaccine type and the chosen vaccination strategy to comprehensively assess cost-effectiveness.

## Figures and Tables

**Table 1:** Input parameters of cost-effectiveness analysis model.

Model Input	Value (EUR)	DSA Range	PSA Distribution	PSA Parameters	Reference
*Unit Costs (Average of All Seasons)*					
*Vaccination costs (fixed)*					
Vaccine per dose	5.79				Boccalini et al., 2018 [28]; EUR of 2017
Administration per dose	10.00				Boccalini et al., 2018 [28]; EUR of 2017
*Direct healthcare costs of Influenza (variable)*					
Pediatrician visit	20.66		gamma (*μ*, *σ*)	(20.66; 2.64)	Ministry of Health [25]; Gross annual salary divided by the number of visits; EUR of 2012
Pharmaceuticals	17.92	2.01–194.02	gamma (*μ*, *σ*)	(17.92; 2.29)	Estimation; EUR of the corresponding observed year
Tests/Examinations	11.14	2.0–51.65	gamma (*μ*, *σ*)	(11.14; 1.42)	Estimation; EUR of the corresponding observed year
Specialist Visit	17.59	5.0–63.9	gamma (*μ*, *σ*)	(17.59; 2.24)	Estimation; EUR of the corresponding observed year
ER visit	22.50		gamma (*μ*, *σ*)	(22.5; 2.87)	Mean; EUR of the corresponding observed year
Hospitalization	3056.27		gamma (*μ*, *σ*)	(3056.27; 0.55)	Pitrelli 2016 [29]; EUR of 2016
Unit Quality-adjust Life Years (QALYs)					
Life Expectancy	79.87				Istat
Utility	0.95		Beta (*α*, *β*)	(8.075; 76.96)	Scalone et al., 2015 [27]
QALY loss from Influenza and Pediatrician visit	0.0090		Beta (*α*, *β*)	(81; 9010.31)	Hollmann et al., 2013 [26]
QALY loss from Influenza and Specialist Visit	0.0112		Beta (*α*, *β*)	(1.229; 108.56)	Hollmann et al., 2013 [26]
QALY loss from Influenza and Hospitalization	0.0430		Beta (*α*, *β*)	(17.627; 392.43)	Hollmann et al., 2013 [26]
*Probabilities (Average of All Seasons)*					
Probability of Influenza (with pediatric visit) if Vaccinated	0.0547	±25%			Estimation
Probability of Influenza (with pediatric visit) if Not Vaccinated	0.0929	±25%			Estimation
Probability of Additional Specialist Visits due to Influenza if Vaccinated	0.0335	±25%			Estimation
Probability of Additional Specialist Visits due to Influenza if Not Vaccinated	0.0258	±25%			Estimation
Probability of Additional Hospital Visits due to Influenza if Vaccinated	0.0049	±25%			Estimation
Probability of Additional Hospital Visits due to Influenza if Not Vaccinated	0.0027	±25%			Estimation

Abbreviation: DSA, deterministic sensitivity analysis; PSA, probabilistic sensitivity analysis.

**Table 2:** Unit costs associated with Influenza (no discount rate measured in EUR.

Season		Unit Costs of Influenza Episodes							Expected Unit Costs	Incremental Cost
		Number of Influenza Cases (n)	Mean Cost (If Influenza)	Median Cost	Interquartile Range (IQR)	Standard Deviation (SD)	Min Cost	Max Cost	Expected Healthcare Expenditure	
All Seasons	Not Vaccinated	17,724	49.10	41.32	(20.66, 60.08)	84.41	20.66	3250.08	4.39	13.44
	Vaccinated	1016	87.21	71.21	(43.77, 92.85)	145.43	36.45	3190.07	17.82	
2009–2010	Not Vaccinated	2473	49.77	41.32	(20.66, 61.98)	94.95	20.66	3250.08	6.02	16.38
	Vaccinated	416	87.21	71.21	(44.32, 98.2)	159.97	36.45	3190.07	22.40	
2010–2011	Not Vaccinated	2794	53.26	41.32	(20.66, 61.98)	106.13	20.66	3141.63	6.78	12.15
	Vaccinated	166	76.99	59.60	(44.13, 91.79)	50.60	36.45	420.69	18.93	
2011–2012	Not Vaccinated	1775	47.57	41.32	(20.66, 58.44)	77.66	20.66	3076.92	3.53	13.88
	Vaccinated	94	69.76	60.72	(42.08, 82.77)	35.70	36.45	213.54	17.41	
2012–2013	Not Vaccinated	2520	46.47	41.32	(20.66, 58.54)	30.97	20.66	438.85	5.77	12.00
	Vaccinated	80	71.89	61.39	(40.88, 86.575)	37.56	36.45	211.71	17.77	
2013–2014	Not Vaccinated	846	60.66	41.32	(20.66, 61.98)	189.54	20.66	3211.22	2.47	14.13
	Vaccinated	34	72.10	63.90	(36.45, 92.91)	36.41	36.45	141.06	16.60	
2014–2015	Not Vaccinated	1753	46.76	38.89	(20.66, 56.35)	80.66	20.66	3188.39	4.56	12.79
	Vaccinated	51	80.66	71.64	(50.98, 98.29)	41.99	36.45	236.6	17.35	
2015–2016	Not Vaccinated	1589	45.17	38.01	(20.66, 55.85)	32.60	20.66	388.23	5.08	12.24
	Vaccinated	53	74.38	68.40	(36.45, 98.43)	33.03	36.45	164.87	17.33	
2016–2017	Not Vaccinated	1117	44.50	37.01	(20.66, 54.92)	31.86	20.66	390.61	3.39	13.18
	Vaccinated	32	166.59	61.88	(36.45, 84.195)	553.14	36.45	3191.67	16.57	
2017–2018	Not Vaccinated	1598	49.09	41.32	(20.66, 60)	82.58	20.66	3076.93	5.67	11.16
	Vaccinated	36	70.78	57.11	(36.45, 86.84)	41.49	36.45	186.77	16.83	
2018–2019	Not Vaccinated	1259	50.49	41.32	(20.66, 58.87)	54.32	20.66	950.73	3.75	13.57
	Vaccinated	54	77.44	65.90	(49.12, 89.79)	58.52	36.45	430.56	17.32	

**Table 3:** Unit health impact of influenza vaccine compared to no vaccination for children in Italy, per patient. Pedianet 2009–2019.

Season		Expected QALY	Incremental QALY Gain
All Seasons	Not Vaccinated	0.94918	0.000451
	Vaccinated	0.94963	
2009–2010	Not Vaccinated	0.94890	0.000006
	Vaccinated	0.94890	
2010–2011	Not Vaccinated	0.94883	0.000525
	Vaccinated	0.94936	
2011–2012	Not Vaccinated	0.94932	0.000286
	Vaccinated	0.94961	
2012–2013	Not Vaccinated	0.94888	0.000680
	Vaccinated	0.94956	
2013–2014	Not Vaccinated	0.94962	0.000196
	Vaccinated	0.94982	
2014–2015	Not Vaccinated	0.94917	0.000488
	Vaccinated	0.94966	
2015–2016	Not Vaccinated	0.94909	0.000549
	Vaccinated	0.94964	
2016–2017	Not Vaccinated	0.94942	0.000361
	Vaccinated	0.94978	
2017–2018	Not Vaccinated	0.94895	0.000812
	Vaccinated	0.94976	
2018–2019	Not Vaccinated	0.94930	0.000379
	Vaccinated	0.94968	

## Data Availability

The data used in this study cannot be made publicly available due to Italian data protection laws. The anonymized datasets generated during and/or analyzed during the current study can be provided on request, from the corresponding author, after written approval by the Internal Scientific Committee (info@pedianet.it).

## Supplementary Materials

The following supporting information can be downloaded at: https://www.mdpi.com/article/10.3390/vaccines12101113/s1, Table S1: Seasonal Vaccine Effectiveness Rates using Risk adjusted Model; Table S2: Matching Table; Table S3: Medicine Used for Influenza Treatment; Table S4: ICER and Cost per Influenza Case Averted (€).
