# Supplementary material for: Cost-Effectiveness of Influenza Vaccination in Healthy Children: A 10-Year Population-Based Study
Source: Vaccines (Basel). 2024 Sep 28;12(10):1113. doi: 10.3390/vaccines12101113 (PMC11511569; doi:10.3390/vaccines12101113)
Supplement: Supplementary file 1 [file vaccines-12-01113-s001.zip › vaccines-3195441-supplementary.pdf]

**Supplementary Materials**

# Cost-Effectiveness of Influenza Vaccination in Healthy Children: A 10-Year Population-Based Study

Elisa Barbieri *, Yuxi Wang, Anna Cantarutti , Antonio Scamarcia , Luigi Cantarutti , Giovanni Corrao, Aleksandra Torbica and Carlo Giaquinto

* Correspondence: elisa.barbieri@unipd.it

## Supplementary methods

### *Complications of interest*

According to SNOMED 2021 guideline a complication is defined as a disorder caused by a procedure or event which is neither a natural progression nor an expected outcome of its cause. In our case complications will be identified during the influenza episode (i.e., within 30 days from the episode starting date).

Complications of interest includes

- Dehydration (only for children up to 24 months of age)
- Febrile seizures
- Neurological complications
- Acute myositis
- Acute myocarditis
- Pneumonia
- Acute otitis media
- Acute sinusitis

All complications were considered possibly associated to the influenza episode if occurring within 30 days from the influenza episode index date.

Dehydration event was retrieved from Pedianet using ICD9-CM code (276.5) or free text based on visit for dehydration in outpatient setting, hospital admission or ER visit where dehydration is one of the diagnosis or where there was a claim for parental re-hydration (i.e. NaCl solution) in the same timeframe of the influenza episode

Febrile seizures were defined as patients with non-focal seizures in the setting of fever who were between 6 months to 6 years of age with no underlying seizure disorder, brain pathology, or significant metabolic disturbance [Newland J et al, 2007]. The event were retrieved from Pedianet using ICD9-CM code (780.31, 780.32) or free text based on visit for febrile seizure in outpatient setting, hospital admission or ER visit where febrile seizure is one of the diagnosis.

Neurological complications were defined as symptoms or signs that affected the central or peripheral nervous system [Newland J et al, 2007] including

- Influenza-related encephalopathy (on the basis of definitions from the Centers for Disease Control )—patients who have had altered mental status or personality change lasting more than 24 hours and occurring within 5 days of onset of the influenza-confirmed event. The event will be retrieved from Pedianet using ICD9-CM code (013.6, 036.1, 046.2, 052.0, 054.3, 055.0, 058.21, 058.29, 062, 063, 064, 066.41, 072.2, 090.41, 094.81, 130.0, 323.01, 323.1, 323.2, 323.41, 323.51, 323.61, 323.62, 323.71, 323.81, 323.9, ) or free text based on visit for influenza-related encephalopathy in outpatient setting, hospital admission or ER visit where influenza-related encephalopathy is one of the diagnosis.

- Post-infectious influenza encephalopathy—patients who have had altered mental status or personality change lasting more than 24 hours and starting more than 5 days after the onset of respiratory symptoms. The event will be retrieved from Pedianet using ICD9-CM code (046.3, 348.30, 348.31, 348.29) or free text based on visit for post-infectious influenza encephalopathy in outpatient setting, hospital admission or ER visit where post-infectious influenza encephalopathy is one of the diagnosis.

- Seizure with fever—patients without an underlying seizure disorder, brain pathology, or significant metabolic disturbance who had a fever and suffered a focal seizure and are less than 6 months old or more than 6 years old. The event will be retrieved from Pedianet using ICD9-CM code (780.39) or free text based on visit for seizure with fever in outpatient setting, hospital admission or ER visit where seizure with fever is one of the diagnosis.

- Other neurologic complications—including Reye syndrome, Guillaine-Barré syndrome and will be retrieved from Pedianet using ICD9-CM code (331.81, 357.0 ) or free text based on visit for neurological disfunction in outpatient setting, hospital admission or ER visit where neurological dysfunction is one of the diagnosis.

Acute myositis events were retrieved from Pedianet using ICD9-CM code (729.1) or free text based on visit for myositis in the outpatient setting and hospital admission or ER visit where myositis is one of the diagnosis.

Acute myocarditis event were retrieved from Pedianet using ICD9-CM code (422.0) or free text based on visit for myocarditis in the outpatient setting and hospital admission or ER visit where myocarditis is one of the diagnosis.

Pneumonia event were retrieved from Pedianet using ICD9-CM code (480, 481,482,483,485,486,487.0) or free text based on visit for pneumonia in the outpatient setting and hospital admission or ER visit where pneumonia is one of the diagnosis.

Suppurative and nonsuppurative otitis media and mastoiditis event were retrieved from Pedianet using ICD9-CM code (381-382, 383) or free text based on visit for otitis in the outpatient setting and hospital admission or ER visit where otitis or mastoiditis is one of the diagnosis.

Acute sinusitis event were retrieved from Pedianet using ICD9-CM code (461) or free text based on visit for acute sinusitis in the outpatient setting and hospital admission or ER visit where acute sinusitis is one of the diagnosis.

Death event occurring up to 30 days after the influenza episode index date and not defined as suicide or sudden death not related to medical condition, were related to the influenza episode. The events were retrieved from the clinical diary field.

## Tables

**Table S1. Seasonal Vaccine Effectiveness Rates using Risk adjusted Model.**

| **Season** | **Hazard Ratio** | **Confidence Interval** | **Vaccine Effectiveness** |
|---|---|---|---|
| 2009-2010 | 0.84 | (0.75 - 0.94) | 16% |
| 2010-2011 | 0.42 | (0.36 - 0.49) | 58% |
| 2011-2012 | 0.52 | (0.42 - 0.64) | 48% |
| 2012-2013 | 0.34 | (0.27 - 0.43) | 66% |
| 2013-2014 | 0.45 | (0.32 - 0.64) | 55% |
| 2014-2015 | 0.49 | (0.37 - 0.65) | 51% |
| 2015-2016 | 0.32 | (0.24 - 0.42) | 68% |
| 2016-2017 | 0.27 | (0.19 - 0.39) | 73% |
| 2017-2018 | 0.30 | (0.21 - 0.41) | 71% |
| 2018-2019 | 0.58 | (0.44 - 0.76) | 43% |

**Table S2. Matching Table.**

| Season | A/H1N1 | A/H3N2 | B | **total** |
|---|---|---|---|---|
| 2009-2010 | U | . | M | **U** |
| 2010-2011 | M | M | M | **M** |
| 2011-2012 | M | M | U | **M** |
| 2012-2013 | M | M | M | **M** |
| 2013-2014 | M | M | M | **M** |
| 2014-2015 | M | U | M | **U** |
| 2015-2016 | . | . | . | **M** |
| 2016-2017 | M | U | . | **.** |
| 2017-2018 | M | U | . | **.** |
| 2018-2019 | M | M | U | **M** |

**Table S3. Medicine Used for Influenza Treatment.**

| Group | ATC |
| --- | --- |
| Antibiotics | J01xxx |
| Amoxicillin | J01CA04 |
| Amoxicillin and clavulanic acid | J01CR02 |
| Cephalosporins | J01Dxx |
| Macrolides | J01FAxx |
| Other antibiotics | J01xxx not included in the previous categories |
| Antivirals | J05xxx |
| Beta2-agonists (nebulized) | R03Axx |
| Glucocorticoids (nebulized) | R03BAxx |
| Other respiratory drugs | R03xxx |

**Table S4.** ICER and Cost per Influenza Case Averted (€).

| | ICER<br>(EUR per QALY) | Cost per Averted Influenza Case (EUR) |
|---|---|---|
| Average (ex 2009-2010) | 29,831 | 23 |
| 2009-2010 | 2,804,180 | 102 |
| 2010-2011 | 23,148 | 21 |
| 2011-2012 | 48,443 | 29 |
| 2012-2013 | 17,653 | 18 |
| 2013-2014 | 72,153 | 26 |
| 2014-2015 | 26,219 | 25 |
| 2015-2016 | 22,285 | 18 |
| 2016-2017 | 36,523 | 18 |
| 2017-2018 | 13,736 | 16 |
| 2018-2019 | 35,803 | 32 |
